# Supplementary material for: Durability of DNA-LNP and mRNA-LNP vaccine-induced immunity against SARS-CoV-2 XBB.1.5
Source: NPJ Vaccines. 2026 Jan 31;11:115. doi: 10.1038/s41541-026-01382-3 (PMC13260325; doi:10.1038/s41541-026-01382-3)
Supplement: Supplementary file 1 — Supplementary Information [file 41541_2026_1382_MOESM1_ESM.pdf]

Supplementary Information

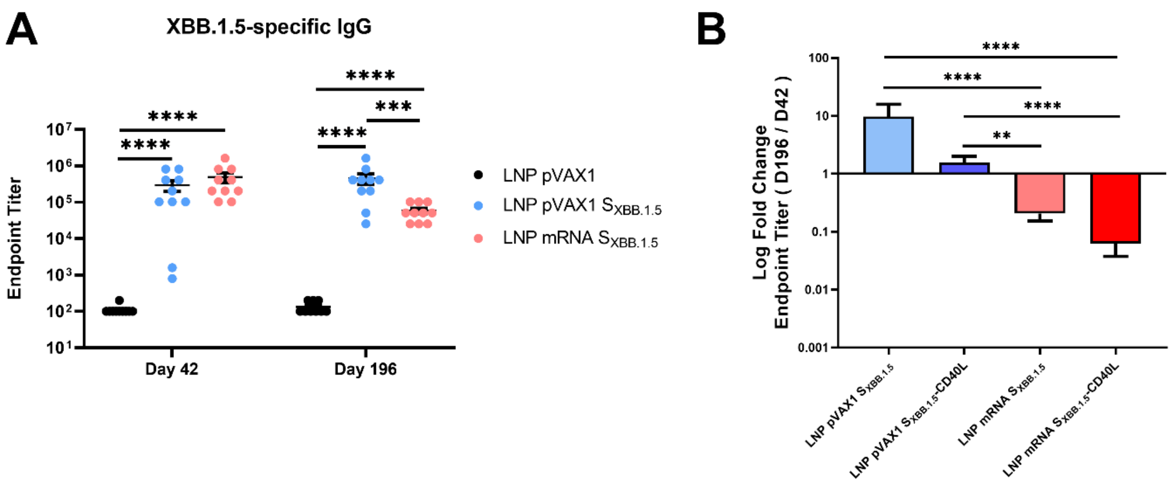

Supplementary Figure 1. Longevity of DNA-LNP immunity is independent of CD40L. (A) Male Syrian hamsters were immunized intramuscularly on day 0 and 28 with 5 µg of $S_{XBB.1.5}$ DNA-LNP or 2.5 µg of $S_{XBB.1.5}$ mRNA-LNP. Control hamsters were vaccinated with 5 µg of pVAX1 DNA-LNP. XBB.1.5 Spike-specific IgG in the serum was determined by ELISA on day 42 or 196 post-vaccination. **(B)** Log fold change in endpoint titers between days 42 and 196 (D196/D42) for $S_{XBB.1.5}$ and $S_{XBB.1.5}$-CD40L (See Figure 2) vaccines. Data shown are mean ± SEM, n= 10. $**p < 0.01$, $***p < 0.001$, $****p < 0.0001$.

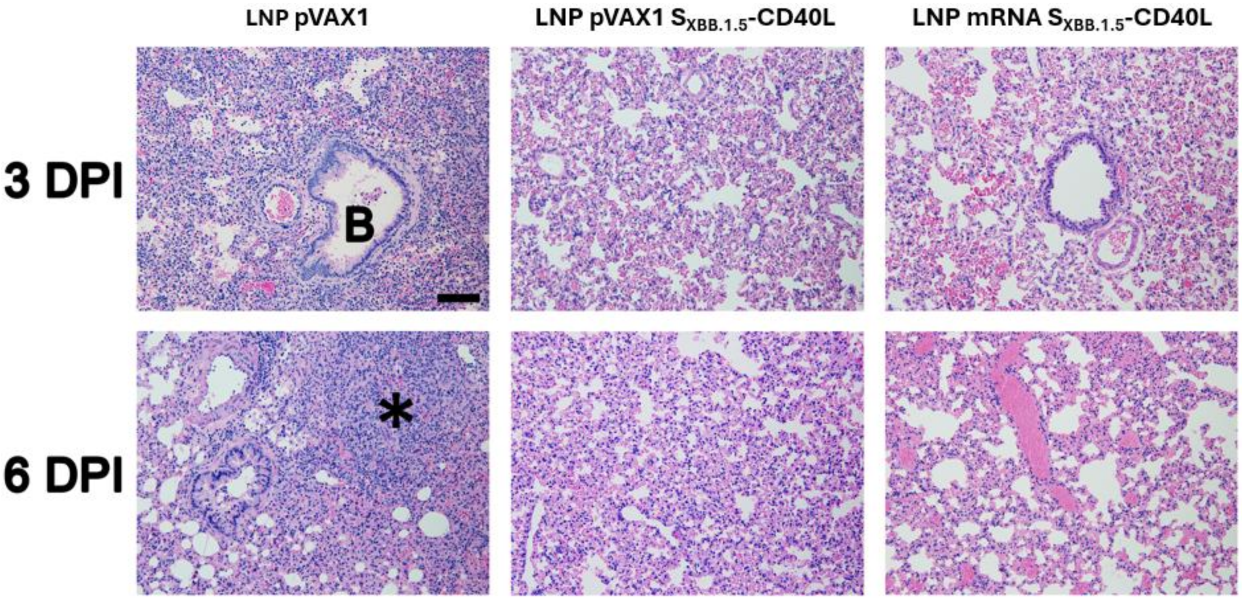

Supplementary Figure 2. Short-Term Lung Pathology. Representative photomicrograph of H&E stained lung tissues. Immunized Syrian hamsters were intranasally challenged with an isolate of SARS-CoV-2 XBB.1.5 on day 49. B, bronchioles; *, area of inflammatory cell infiltration and tissue consolidation. Scale bar, 100 µm. Related to Figure 3.

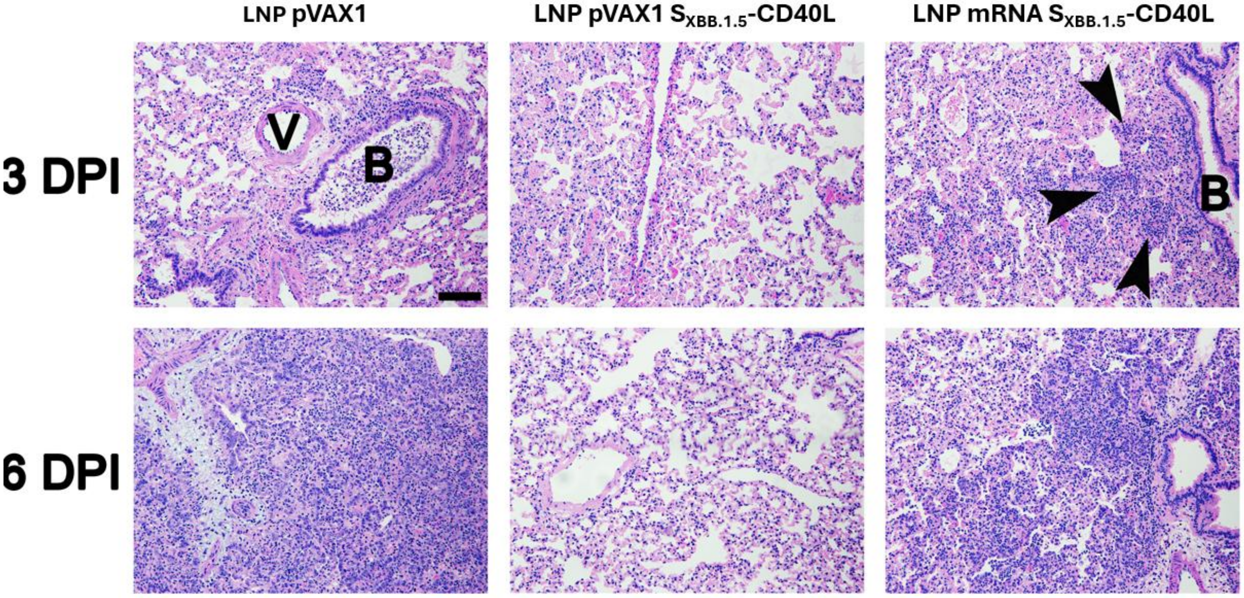

Supplementary Figure 3. High Magnification Long-Term Lung Pathology. Representative photomicrograph of H&E-stained lung tissues. Immunized Syrian hamsters were intranasally challenged with an isolate of SARS-CoV-2 XBB.1.5 on day 203. B, exudate of inflammatory cells in the lumen of a bronchus; V, blood vessel. Arrows, peri-airway inflammatory cell infiltration and tissue consolidation. Scale bar, 100 µm. Related to Figure 4.

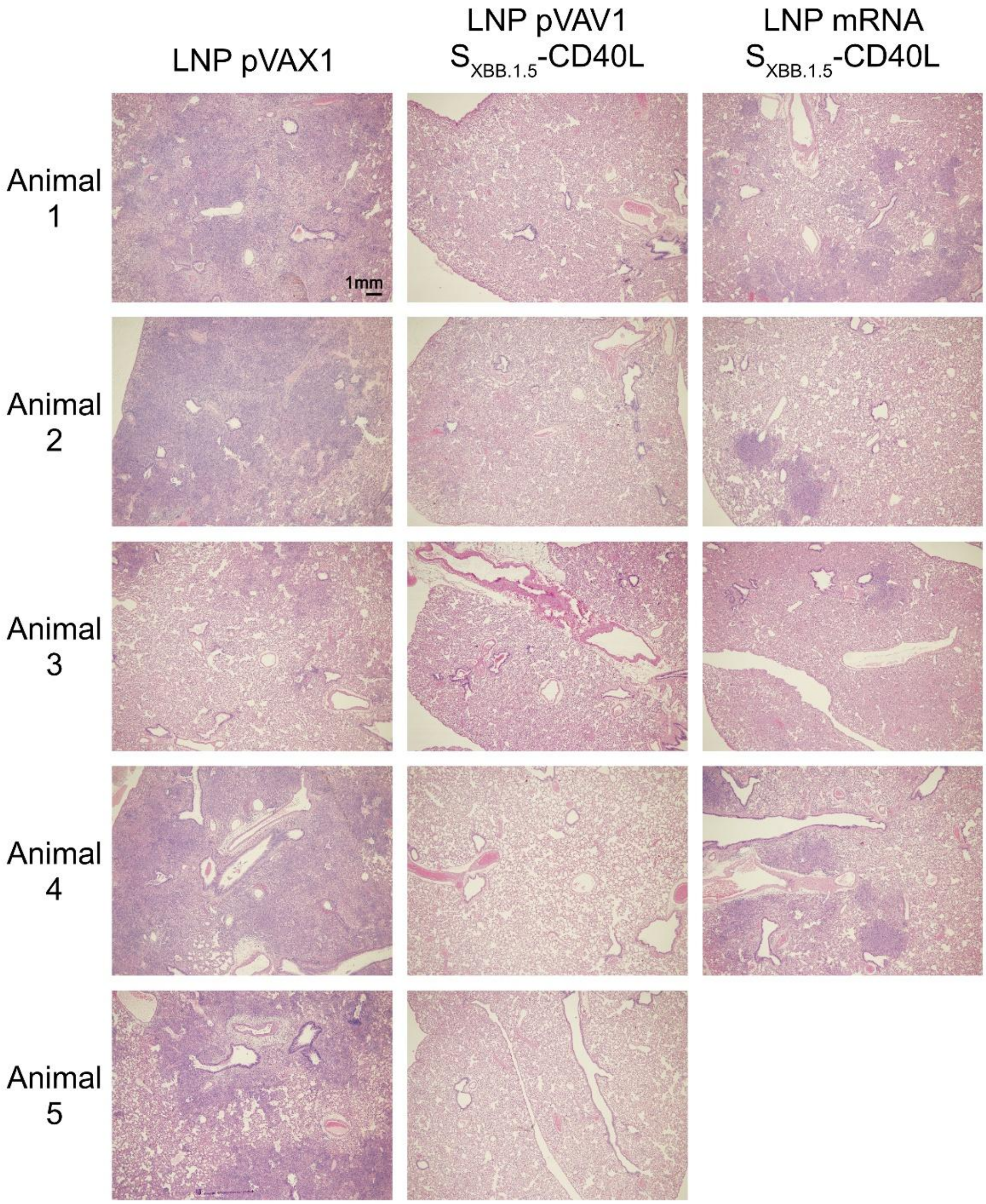

Supplementary Figure 4. Low Magnification Long-Term Lung Pathology. Representative photomicrograph of H&E-stained lung tissues at 1X magnification, demonstrating global lung morphology at 6-dpi. Immunized Syrian hamsters were intranasally challenged with an isolate of SARS-CoV-2 XBB.1.5 on day 203. Related to Figure 4.

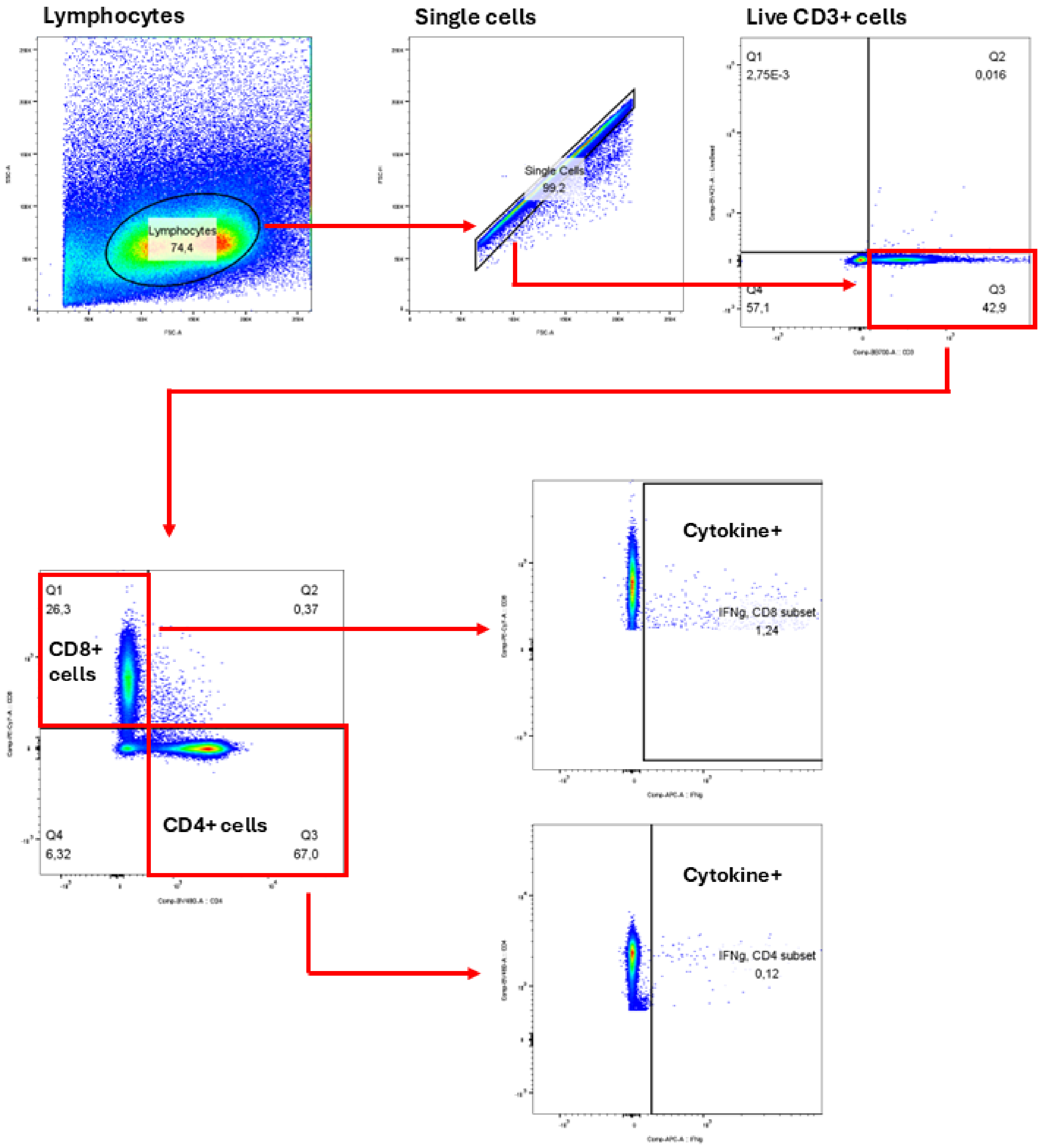

Supplementary Figure 5. T cell gating strategy. Gating strategy for cytokine expression in $CD4^+$ and $CD8^+$ T cells isolated from BALB/c spleens. Representative plots from a $S_{XBB.1.5}$-CD40L mRNA-LNPs vaccinated mouse. Related to Figure 1.

Supplementary Table 1. Long-Term Lung Pathology Per Lobe. Immunized Syrian hamsters were intranasally challenged with an isolate of SARS-CoV-2 XBB.1.5 on day 203. At 6-dpi, the severity and extent of pneumonia was assessed independently for each right lung lobe (labeled arbitrarily from A-D). Related to Figure 4.

	**LNP pVAX1**					**LNP pVAX1 $S_{XBB.1.5}$-CD40L**					**LNP mRNA $S_{XBB.1.5}$-CD40L**			
**Lobe**	**1**	**2**	**3**	**4**	**5**	**1**	**2**	**3**	**4**	**5**	**1**	**2**	**3**	**4**
**A**	4	4	0.5	4	4	0	0	0	0	0	4	2.5	1.5	3
**B**	4	4	3	4	4	0	0	0	0	0	3.5	3	1.5	3
**C**	4	4	2.5	3	4	0	0	0	0	0	2	1	2	3
**D**	3	-	-	4	3	0	0	0	-	0	0	-	2	3

Supplementary Table 2. Characterization of mouse DNA- and mRNA-LNP vaccines. Biophysical characterization parameters of DNA- and mRNA-LNP vaccines used in BALB/c studies. Mean particle diameter measured by nanoparticle tracking analysis (NTA). Encapsulation efficiency determined by SYBR™ Gold assay. NT, nucleotides. BP, base pairs. SD, standard deviation. Related to Figure 1.

Vaccine (length, NT or BP)	Prime/Boost	LNP Diameter (Mean ± SD , nm)	Encapsulation efficiency (%)
pVAX1 (2999)	Prime	67.5 ± 10.7	97.6
	Boost	69.1 ± 12.1	96.0
mRNA $S_{XBB.1.5}$ (3659)	Prime	69.2 ± 13.0	88.5
	Boost	74.0 ± 14.9	86.0
mRNA $S_{XBB.1.5}$-CD40L (4102)	Prime	72.2 ± 14.8	83.9
	Boost	75.4 ± 17.6	80.7

Supplementary Table 3. Characterization of hamster DNA- and mRNA-LNP vaccines. Biophysical characterization parameters of DNA- and mRNA-LNP vaccines used in Syrian hamster studies. Mean particle diameter measured by NTA. Encapsulation efficiency determined by SYBR™ Gold assay. NT, nucleotides. BP, base pairs. SD, standard deviation. Related to Figure 2-4 and Supplementary Figure 1.

**Vaccine (length, NT or BP)**	**Prime/Boost**	**LNP Diameter (Mean ± SD , nm)**	**Encapsulation efficiency (%)**
pVAX1 (2999)	Prime	72.2 ± 14.6	93.4
	Boost	71.3 ± 14.2	94.0
pVAX1 $S_{XBB.1.5}$ (6658)	Prime	79.7 ± 18.2	92.4
	Boost	78.5 ± 18.7	91.8
pVAX1 $S_{XBB.1.5}$-CD40L (7101)	Prime	81.3 ± 19.5	94.3
	Boost	81.7 ± 20.9	93.1
mRNA $S_{XBB.1.5}$ (3659)	Prime	71.6 ± 15.2	83.8
	Boost	71.7 ± 13.3	88.4
mRNA $S_{XBB.1.5}$-CD40L (4102)	Prime	72.2 ± 16.7	80.7
	Boost	72.2 ± 16.1	85.2
